# Annotations

**Published:** 1891-11-07

**Authors:** 


### Annotations.

Unpractical London.

Not so very long ago London spent over £4,000,000 on its main drainage work. It is now proposed to spend over £2,000,000 more. If the money were to be spent on works which would rid us of our sewage without poisoning our river, none of us would grudge the £2,000,000, or indeed ten times that amount. But the proposal is one for merely increasing the capacity of our present drainage system. It is a feeble proposal, and a counsel of despair. At present, as everybody knows but too well, the Thames at certain hours of the day is merely an open drain. The writer crossed London Bridge a week or two ago, on a somewhat warm afternoon. The water was low and the tide just on the turn; the look of the river was black, filthy, and horrible; the smell was overpowering, poisonous, deadly. From one side of the bridge to the other, and for a long distance on each bank the air was pestiferous. No practical man crossing the river at such a time could hold a high opinion of the engineers and doctors of London. Our method of disposing of the sewage is worthy of the savages of Central Africa. Three thousand years ago Moses would have devised a ten times better system. We, in London are but children in our capacity for dealing with sewage; we have under our feet eighty two miles of main sewers; we throw into the river every day a hundred and eighty million gallons of liquid poison. Much of what is poisonous in the river would be of great money value on the land. Nobody who really understands anything practically about the subject denies this. A few town-bred engineers and laboratory chemists may speak otherwise; but their opinions are not worth the breath that is expended in uttering them. "The sewage to the soil" is the decisive judgment of those who understand what they are talking about. Tunbridge Wells is one of the healthiest towns in England, and one reason is that it has no river into which to eject its sewage, and it has, therefore, been compelled to get rid of it by other means. It has two sewage farms, both of which are paying substantial interest on invested capital. The same is true of Edinburgh. Edinburgh could, with much more reason, throw its sewage into the Firth of Forth than London can empty its vile drains into the Thames. But Edinburgh knows better; its citizens have brains; brains and public spirit. London is a poor place for public spirit and intellectual capacity. What is to prevent us having a hundred sewage farms within a radius of fifty miles of Charing Cross? Of course a single writer on a subject like this feels that he is like a "voice crying in the wilderness." But if he knows that he is right it is his duty to persist in crying all the same, and he makes up his mind that he will continue to do so as long as he can hold a pen and command the help of a publisher.

Science, Religion, and Mariana in her Moated Grange.

England swarms with men of science, and with priests and parsons of all sorts. The one class holds up sanitary ideals, and displays an infinite variety of laboratory microbes for our admiration; the other preaches moral perfection Sunday by Sunday, and not seldom during the week. This teaching and preaching of science and religion has been going on for an indefinite time. Moreover, the clergy never tire of exhorting the laity to empty their pockets into the missionary box. One large missionary society is actually proposing to send out a hundred additional European missionaries to foreign parts, although it cannot meet its current expenses without great difficulty. Now, is it possible to persuade some of our men of science and clergymen to lay aside for a few minutes their microscopes and their microbecultivations, their surplices and their manuscript sermons, and to give half-an-hour's thought to Quainton, near Aylesbury, and some of its neighbour villages, places within a mere afternoon cycle-ride of London? According to a correspondent of the *Daily News*, the man of science and the clergyman who should condescend to spend a half-holiday in those villages would find an epidemic of diphtheria prevailing. The disease has already attacked thirty-two persons of the small population, and has killed thirteen of them, almost one in every two attacked; a truly terrible mortality. The medical officer for the Aylesbury rural sanitary district has just submitted a report to his local superiors. He found that the epidemic began in a certain house. The particular house has no trap to its drain, and its closet opens directly into the sewer. That was surely bad enough, but it was almost sanitary perfection compared with the next house which was examined. It also was a cottage. It had an open gutter three feet from its back door, and the gutter ran into an untrapped drain. Immediately facing the back door, and on a higher level, were five pig-sties. The poisonous filth from the five sties ran down into the gutter at the back door, and still runs down for anything we know to the contrary. Some of our readers must be aware of the abominable odour of a pig-sty in hot weather, or, indeed, in any weather. But what must it be to have five pig-sties close to your door, your window, your bed-room window, under your very nose in fact, day and night? Two people in this cottage took diphtheria, and one died. The next cottage has a thatched roof which admits the rain. The sink discharges itself upon the ground close to the foundation, and the putrid matter has been percolating daily into the foundations of the house for years, perhaps for centuries. The next cottage kindly sends on the contents of its sink through a little drain which empties itself also into the foundations of its neighbour's house. Near these delightful abodes is, of all things in the world, a dairy. The medical officer visited three other cottages, each of which was actually surrounded by a filthy cesspool: three "Moated Granges" of the era of the nineteenth century! One of these "Moated Granges" is occupied by a "Mariana" with six children. She has only one bedroom, but the doctor believes no beds at all. The whole family sleep on the bare boards. Three of the inhabitants of one house have already died. The epidemic is still in full swing, six cases have been removed to the hospital, and two of them are critical. This is the state of some of the "Cottage Homes of England," of which an enthusiastic poet sings—"How beautiful they stand"? They certainly do. One cannot help admiring them, and their inhabitants, and their proprietors. If anything can make an Englishman feel disgraced in his very soul, it is facts of this kind. How can one help a feeling of indignant scorn against the parson, the doctor, the proprietor of the cottages, and even against the wretched inhabitants themselves? There seems to be neither public spirit nor private sense in Aylesbury or its neighbourhood. Are there any more such villages in England, and near its "Stately Homes"? If there are let the newspapers send out an army of correspondents to tell us of them; and let us see if it is possible for us to feel as much shame as will bring us to a determined effort to sweep such things away, at once and for ever.

Surgeon Parke's Book.

Surgeon Parke, in his record of his African experiences with Stanley, just published, has shed a little kindly light, not only on Darkest Africa, but on some of its illuminators. And the light was much needed. Africa seems to spoil the tempers of all sorts of people. Even the missionaries of the various denominations, who eat with one another as they travel up and down the dark continent, often traduce one another very elaborately when they return home. The members of the Stanley expedition seem to have been no exceptions to this temper-spoiling rule. How they "raged" against one another after their return home, like "the heathen" with whom the psalmist was displeased, is known to everybody. They appeared to be almost all possessed with the same eager desire to fly at each other's throats. If they behaved to one another in Africa as they did in England the untutored savages might well exclaim: "See how those Christians hate one another." Surgeon Parke, by his book, does something to redeem the character of Stanley and his fellow explorers. He speaks kindly and generously of them all. His book is a wonderful record of travel, of strenuousness, of almost superhuman difficulties overcome by more than human courage and resolution. It deals much with travel and little with medicine and medical discovery. Surgeon Parke had small opportunity for testing native remedies. His chief business was to keep his fellow travellers and himself alive. The providing of food and the minimising of the effects of daily accidents were therefore his most urgent care. The tone of the book reveals a man who is accustomed to measure his words and to express his ideas with scientific accuracy and precision. Such a book is distinct evidence of the value of medica training. Surgeon Parke learnt at his medical school to make his language the just measure of the facts. Exaggeration and "flamboyancy" of all kinds are hateful to the scientific medical mind, and should be equally hateful to all those who write for the public in any and every capacity.

